# Knowledge-Infused Abstractive Summarization of Clinical Diagnostic Interviews: Framework Development Study

**DOI:** 10.2196/20865

**Published:** 2021-05-10

**Authors:** Gaur Manas, Vamsi Aribandi, Ugur Kursuncu, Amanuel Alambo, Valerie L Shalin, Krishnaprasad Thirunarayan, Jonathan Beich, Meera Narasimhan, Amit Sheth

**Affiliations:** 1 Artificial Intelligence Institute University of South Carolina Columbia, SC United States; 2 Kno.e.sis Center Department of Computer Science and Engineering Wright State University Dayton, OH United States; 3 Department of Psychology Wright State University Dayton, OH United States; 4 Department of Psychiatry Wright State University Dayton, OH United States; 5 Department of Neuropsychiatry & Behavioral Science School of Medicine, Prisma Health University of South Carolina Columbia, SC United States

**Keywords:** knowledge-infusion, abstractive summarization, distress clinical diagnostic interviews, Patient Health Questionnaire-9, healthcare informatics, interpretable evaluations

## Abstract

**Background:**

In clinical diagnostic interviews, mental health professionals (MHPs) implement a care practice that involves asking open questions (eg, “What do you want from your life?” “What have you tried before to bring change in your life?”) while listening empathetically to patients. During these interviews, MHPs attempted to build a trusting human-centered relationship while collecting data necessary for professional medical and psychiatric care. Often, because of the social stigma of mental health disorders, patient discomfort in discussing their presenting problem may add additional complexities and nuances to the language they use, that is, hidden signals among noisy content. Therefore, a focused, well-formed, and elaborative summary of clinical interviews is critical to MHPs in making informed decisions by enabling a more profound exploration of a patient’s behavior, especially when it endangers life.

**Objective:**

The aim of this study is to propose an unsupervised, knowledge-infused abstractive summarization (KiAS) approach that generates summaries to enable MHPs to perform a well-informed follow-up with patients to improve the existing summarization methods built on frequency heuristics by creating more informative summaries.

**Methods:**

Our approach incorporated domain knowledge from the Patient Health Questionnaire-9 lexicon into an integer linear programming framework that optimizes linguistic quality and informativeness. We used 3 baseline approaches: extractive summarization using the SumBasic algorithm, abstractive summarization using integer linear programming without the infusion of knowledge, and abstraction over extractive summarization to evaluate the performance of KiAS. The capability of KiAS on the Distress Analysis Interview Corpus-Wizard of Oz data set was demonstrated through interpretable qualitative and quantitative evaluations.

**Results:**

KiAS generates summaries (7 sentences on average) that capture informative questions and responses exchanged during long (58 sentences on average), ambiguous, and sparse clinical diagnostic interviews. The summaries generated using KiAS improved upon the 3 baselines by 23.3%, 4.4%, 2.5%, and 2.2% for thematic overlap, Flesch Reading Ease, contextual similarity, and Jensen Shannon divergence, respectively. On the Recall-Oriented Understudy for Gisting Evaluation-2 and Recall-Oriented Understudy for Gisting Evaluation-L metrics, KiAS showed an improvement of 61% and 49%, respectively. We validated the quality of the generated summaries through visual inspection and substantial interrater agreement from MHPs.

**Conclusions:**

Our collaborator MHPs observed the potential utility and significant impact of KiAS in leveraging valuable but voluminous communications that take place outside of normally scheduled clinical appointments. This study shows promise in generating semantically relevant summaries that will help MHPs make informed decisions about patient status.

## Introduction

### Background

The diagnosis of mental illness is unique to medicine. Although other specialties can rely on physical examinations, imaging, and laboratory tests for diagnosis and ongoing assessment, psychiatry often relies on only a patient’s narrative. An accurate assessment, diagnosis, and treatment hinge on the ability of a trained mental health professional (MHP) to elicit not only information but also subtle indicators of human emotions that portend clues to severely life-threatening situations [1]. Although MHPs might find a “second set of eyes or ears” valuable, it is generally impractical and costly to hire additional personnel for this purpose. The shortage of qualified MHPs and the increasing amount of clinical data dictate novel approaches in the diagnosis and treatment processes. Summarizing patients’ relevant electronic health records, including clinical diagnostic interview logs between clinicians and patients, has emerged as a novel method. Simultaneously, the techniques and tools require rigorous evaluation by domain experts [2,3]. As accuracy and false-negative rate are crucial metrics for the success of the deployment of such tools, we leveraged knowledge-infused learning to achieve this goal, as described in recent studies [4-9]. Sheth et al [6,8] define knowledge-infused learning as “the exploitation of domain knowledge and application semantics to enhance existing artificial intelligence methods by infusing relevant conceptual information into a statistical and data-driven computational approach,” which in this study is *integer linear programming* (ILP). A paper on “knowledge infusion” from Valiant et al [10] theoretically assesses the importance of teaching materials (eg, lexicons) in reducing prediction errors and making the model robust. This study, theoretically, quantitatively, and qualitatively evaluates the knowledge-infusion paradigm in improving the outcomes of recent artificial intelligence algorithms, specifically in the context of deep-learning algorithms, as defined in Sheth et al [11,12] on knowledge-infused learning and for achieving explainability.

Building upon the recent efforts in knowledge-infused learning, we propose an end-to-end summarization framework, called KiAS (knowledge-infused abstractive summarization) for clinical diagnostic interviews, using domain knowledge derived from the Patient Health Questionnaire-9 (PHQ-9). Informative summaries capture insightful questions from the interviewer and relevant patient responses that best express intent and expected behavior. This system concisely encapsulates major themes and clarifies patient concerns toward the goal of focused treatment. In addition, summaries for individual patients will provide a new type of historical record of MHP-patient interactions that was not previously possible. This allows for more quantifiable measures for the program of a patient of mental health. We validated our approach by using the *Distress Analysis Interview Corpus-Wizard of Oz* (DAIC-WoZ) data set comprising recorded interviews between a patient (participant) and a computerized animated virtual interviewer *Ellie* [13]. Our MHP coauthors analyzed the clinical intricacies in the data set (described in the *Data Set and Analysis* section). Our analysis of the corpus revealed a key finding: a clinical interview’s patient response is not always specific to the previous question. Instead, a meaningful response from a patient may be semantically linked to other earlier questions. Furthermore, the patient’s responses can be ambiguous, be redundant, and, with distant anaphora, challenge the summarization task.

Previous work has attempted to summarize structured interviews or meeting logs where every response to a question is known to be informative and nonredundant [14]. The dialogues in clinical interviews are ambiguous and, following a simple filtering or preprocessing process, leads to the loss of critical pieces of information that might be relevant to MHPs. Therefore, in our task, we did not consider redundant responses or ambiguous responses as noise. Our aim is to prefer recall over precision because MHPs will eventually decide the action plan and reduce their manual labor.

Abstraction-based summarization is generally more complicated than extractive summarization (ES), as it involves context understanding, content organization, rephrasing, and relevance-based matching of sentences to form coherent summaries. In addition, the challenges in the problem domain increase the complexity of the abstractive summarization (AS) task. Previous studies by Clarke and Lapata [15,16] provided the first attempt for AS using sentence compression techniques (eg, tree based [17] and sentence based [eg, lexicalization or markovization]). However, these approaches rely on syntactic parsing using a part-of-speech tagger, which relies on quality annotation, a process that is both knowledge-intensive and time-consuming [18]. Instead, a direct word graph (WG)–based approach was used employing TextRank to generate compressed sentences by finding the k-shortest paths [19,20]. Filippova used TextRank over LexRank [21] because of the use of a cosine similarity, which is not semantically preserved, a property required in meaningful summarization. However, linguistic quality is sacrificed while improving the informativeness of the summaries. Banerjee et al [22] and Nayeem et al [23] developed an AS scheme using a skip-gram word-embedding model and ILP to summarize multiple documents. Our proposed method optimizes the grammaticality and informativeness constrained by the length of the summaries [22,23].

Recently, researchers have employed a neural network–based framework to address the summarization problem. Li et al [24] described an attention-based encoder and decoder component to find words or phrases that would guide ILP procedures to generate concise and meaningful summaries.

See et al [25] summarized news articles using a supervised sequence-to-sequence approach that uses human-written summaries to learn model parameters. They evaluated their approach using the CNN or Daily Mail data set, which contains news articles and their corresponding human-written abstracts. This method outperformed the state-of-the-art solution by at least two Recall-Oriented Understudy for Gisting Evaluation (ROUGE) points [26]. However, it is not appropriate for summarizing dialogues in diagnostic interviews in an unsupervised setting.

Shang et al [27] designed an unsupervised, end-to-end AS architecture to summarize the meeting speech. However, a meeting structure is different from a clinical interview structure, mostly because it is centered around diagnosing patients. Redundancy in a corpus of interviews is semantic and not merely lexical. An MHP might ask the following 2 paraphrased questions during an interview: (Q1) “Have you been diagnosed with clinical depression?” or (Q2) “You are showing signs of clinical depression; have you seen an MHP before?” Whereas such paraphrasing is absent in a meeting, these questions require domain knowledge to calculate their semantic proximity. Therefore, the approach proposed by Shang et al [27] does not apply to our problem.

Furthermore, the study by Wang et al [28] composed word embedding and word frequency to calculate a *word attraction force score* to integrate previous knowledge. Such word-based models seldom capture implicit semantics (eg, “difficulty in sleeping at night” can allow an MHP to infer insomnia) in complex discourse such as clinical interviews. Identifying relevant phrases (eg, “feeling hopeless”) and characterizing patient behavior is essential for an MHP to subsequently make informed decisions. In addition, word-embedding models (eg, BERT or Word2Vec) do not conflate and provide robustness against lexical ambiguity [29]. Furthermore, the scarcity of clinical diagnostic interviews restricts the training of problem-specific word-embedding models.

MacAvaney et al [30] designed a method to summarize radiology reports using a neural sequence-to-sequence architecture, infusing previous knowledge in the summarization process through ontology. They compared the use of medical ontology (Quick Unified Medical Language System [QuickUMLS]) with domain-specific ontology (RadLex). They evaluated their approach using human-written summaries and acknowledged that AS methods generate readable summaries. For a comprehensive evaluation, human evaluators employed the criteria of readability, accuracy, and completeness. The current state-of-the-art deep-learning architecture, BART (Bidirectional and Auto-Regressive Transformers) [31], comprises a bidirectional encoder over the document to be summarized and an autoregressive decoder over reference summaries (RS). The model is trained using cosine-similarity loss, resulting in a dense representation, which generates summaries using beam search. The method is useful in question answering, reading comprehension, and summarization with gold standard summaries [32,33]. The complexity of the model requires considerable training time over a sizable domain-specific corpus. Unfortunately, most of the previous works on AS have been developed and tested on benchmark data sets on news articles and meeting notes, which differ significantly from clinical interviews. Furthermore, domain knowledge inclusion is critical for associating a meaningful response to the relevant question asked by an MHP, which is challenging for current deep-learning architectures for summarization.

To leverage the benefits of domain knowledge, we used the PHQ-9 lexicon to incorporate relevant concepts in a machine-processable manner. Yazdavar et al [34] built a depression lexicon from the established clinical assessment questionnaire, PHQ-9. They divided the lexicon into 9 signals as per PHQ-9, denoted using indicative phrases as follows: decreased pleasure in most activities (S1), feeling down (S2), sleep disorders (S3), loss of energy (S4), a significant change in appetite (S5), feeling worthless (S6), concentration problems (S7), hyper or lower activity (S8), and suicidal thoughts (S9). Karmen et al [35] also proposed a lexicon for depression symptoms but did not categorize them. Neuman et al [36] crawled the web for metaphorical and nonmetaphorical relations that embed the word *depression*, which added noise to the lexicon because of its polysemous nature (eg, great depression, depressing incident, depressing movie, and economic depression). Furthermore, these lexicons captured *transient sadness* instead of clinical symptoms influencing the diagnosis of *major depressive disorder*. The lexicon created by Yazdavar et al [34] was chosen to conduct this study based on the shortcomings of the alternatives. Alhanai et al [37] used the DAIC-WoZ data set to predict the depression of an individual leveraging multimodal data. The study used audio, visual, and textual data recorded during an interview between a patient and a virtual clinician to train 2 sequence models for detecting depression. Furthermore, the data set was annotated with labels that help train supervised models for detecting depression. However, the DAIC-WoZ data set does not have ground truth summaries that support our research.

### Objective

**Figure 1:**
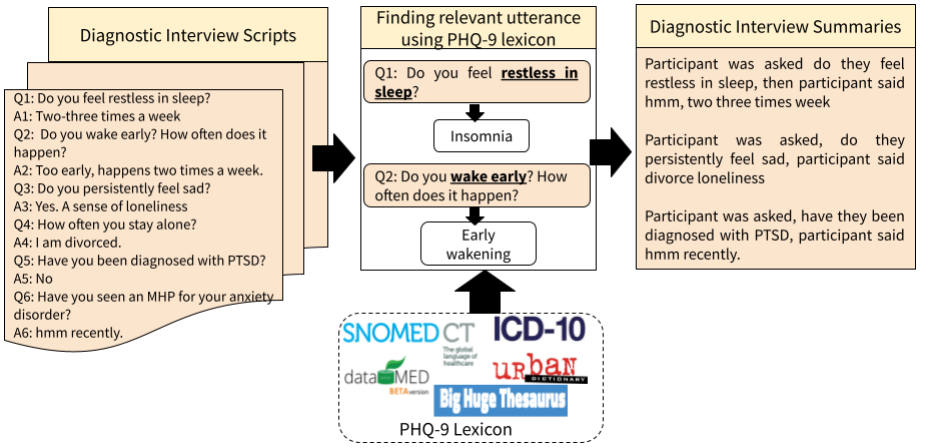
Overview of knowledge-infused abstractive summarization for an interview snippet of a patient's responses to the question asked by Ellie (virtual interviewer). Phrases relevant to mental health are identified using the PHQ-9 lexicon. The contextual similarity between utterances is calculated through a retrofitted embedding model. The resulting summaries contain relevant questions and meaningful responses. ICD-10: International Statistical Classification of Diseases, Tenth Revision; MHP: mental health professional; PHQ-9: Patient Health Questionnaire-9; PTSD: posttraumatic stress disorder; SNOMED CT: SNOMED Clinical Terms.

We seek to improve upon these existing approaches by capturing the critical nuances of clinical diagnostic interviews that will lead to a more robust analysis, paraphrasing, and reorganizing the source content [38]. Our approach uses an ILP framework, exploiting linguistic quality constraints to capture end-user information needs. Furthermore, existing methods seldom leverage domain-specific information to generate summaries by filtering out the noninformative utterances of a patient. For example, the utterance “uh well most recently we went to Israel for a pilgrimage” is informative in everyday conversation but not to an MHP. Furthermore, an isolated utterance “I have trouble sleeping” might not be considered informative by a purely statistical algorithm but is essential information for an MHP. We leveraged a semantic lexicon from a recent study by Yazdavar et al [34] to filter out irrelevant utterances. The lexicon was used to retrofit (contextualize) ConceptNet word embedding and improve the informativeness of summaries [39]. For instance, in Figure 1, the patient replied “No” to the question “Have you been diagnosed with PTSD?” However, the patient replied “hmm recently” to the question “Have you seen an MHP for your anxiety disorder?” During the flattening of the hierarchy of Systematized Nomenclature of Medicine-Clinical Terms (SNOMED-CT) medical knowledge in the semantic lexicon, we observed anxiety disorder (SNOMED-CT ID: 197480006) and posttraumatic stress disorder (PTSD; SNOMED-CT ID: 47505003) were associated with parent-child relationships. Thus, in the generated summary, for the question “Have you been diagnosed with PTSD?” the deduced response is “hmm recently,” which could be a limitation of the summarizer; however, the generated summaries are intended for further scrutiny from MHPs. The proposed approach is illustrated in Figure 1.

A total of 4 professors assessed a set of summaries in neuropsychiatry specializing in rehabilitation counseling and mental health assessment. We avoided crowdsourcing of workers for qualitative evaluation because of issues described in a study by Chandler et al [40]. The key contributions of this study are four-fold:

- We developed an unsupervised framework to generate readable and informative summaries of clinical diagnostic interviews. The proposed KiAS framework does not only rely on target summaries but also uses domain-specific knowledge based on PHQ-9 [4]. The summarization task was formulated to optimize both linguistic quality and informativeness.
- The PHQ-9 lexicon knowledge is infused into KiAS through a trigram language model (LM) and a retrofitted ConceptNet embedding [5,6].
- A quantitative evaluation to assess the efficacy of our approach is based on contextual similarity, Jensen Shannon Divergence (JSD; information entropy), thematic overlap, and readability. As the data set lacks ground truth handwritten summaries, such metrics would evaluate coherence, content-preserving nature, and summaries’ understandability.
- A qualitative evaluation was used to assess the usability of the summaries based on context-specific questions and meaningful responses.

The infusion of distress-related knowledge through language modeling and embedding methods has considerably improved the quality of summaries over comparable baselines. Furthermore, KiAS summarizes long conversations (mean 2061, SD 813 words) in 7-8 sentences (103 words on par with an SD of 52 words) and has the potential to reduce the follow-up time from 3 patients to 5-6 patients in 7 days.

## Methods

### Data Set and Analysis

We used the DAIC-WoZ data set, which consists of clinical interviews to support the diagnosis of psychological disorders such as anxiety, depression, and PTSD [13]. Data were collected via Wizard-of-Oz interviews, conducted by an animated virtual interviewer called Ellie, and controlled by a human interviewer. It contains data from 189 patient interviews, including transcripts, audio recordings, video recordings, and PHQ-8 depression questionnaire responses [41]. For this study, we used only the transcripts. We did not employ existing annotations in the data set in our unsupervised approach. Incomplete words were replaced with their complete version (eg, a vague word such as *peop* is turned into its full form *people*), and unrecognizable words were annotated as *xxx*. The interviews were generally 7-33 minutes long, with an average length of 16 minutes and 58 statements. The data set was anonymized to comply with privacy and ethical standards. From 189 interviews, 5 were excluded from the study either because of imperfections in data collection or transcription (interruptions during the interview or missing transcription of the interviewer’s utterances). Note that we are not predicting depression; instead, we retrieve utterances that help an MHP make inferences relevant to depression, PTSD, or stress.

Our collaborator, clinical psychiatrists, observed that questions related to mental health conditions varied based on their sequence. The clinical diagnostic interviews were open ended and semistructured (eg, with a clinician improvising questions based on an earlier response of a patient) where items transition from clinically irrelevant to clinically relevant (Textbox 1). The interview started with neutral questions to build a rapport with the participant before moving on to inquiries related to depression, PTSD, or mental health. Finally, it ends with a *cool-down* phase to ensure that the participant leaves the interview in a peaceful state of mind. The DAIC-WoZ interviews were designed to diagnose mental health conditions and their severity by assessing the level of interference in a patient’s life. However, much of the meeting is designed to relax the participant by engaging them in neutral conversations. Utterances in a neutral conversation do not provide clinicians with insights about the mental condition; therefore, they can be discarded, and we call this operation *pruning.* Identifying *revealing and diagnostic* utterances is challenging because, without background knowledge, such critical utterances can be missed by statistical methods that are based on frequency counts. For example, if a patient says “nobody likes me” once, a purely statistical approach may not consider this utterance relevant. However, using background knowledge (in our case, the depression lexicon), we can identify such valuable signals. Similarly, the utterance “I was raised in New York, but I live in L.A. now” is irrelevant to a clinician but may be identified as relevant by a domain-agnostic tool. To prune noisy questions or answers and focus on mental health–related issues, we used the PHQ-9 lexicon for semantic pattern-matching of phrases in a conversation. Specifically, semantic pattern-matching captures PHQ-8 responses implicit in the DAIC-WoZ corpus of interviews [42]. On the other hand, previous work on ES relies on the intrinsic capability to remove noisy utterances from conversations instead of a domain-specific lexicon [43] (we provided our evaluation for comparison in the *Results* section). Finally, we converted the questions and their answers from filtered conversations into one combined statement. For example, the question asked: “How long ago were you diagnosed;” patient’s response: “a few years ago”; converted statement: “participant was asked how long ago they were diagnosed,” and the participant replied “a few years ago.”

**Example questions from the interviewer at the start of the interview (top). These questions are casual to make the patient comfortable. Over time, questions become specific to the patient’s condition and behavior (middle). At the end of the interview, the questions become less subjective and target the patient’s life (bottom).**

**Start of interview**

- “Okay what’d you study at school”
- “That’s good where are you from originally”
- “How do you like L.A.”

**Middle of interview**

- “Is there anything you regret”
- “Do you feel down”
- “Have you been diagnosed with posttraumatic stress disorder”

**End of interview**

- “Okay when was the last time you felt really happy”
- “Cool how would your best friend describe you”
- “who’s someone that’s been a positive influence in your life”

We converted the question and answer (Q and A) pairs into sentences to preserve the inherent structure of clinical diagnostic interviews, which is described by the sequence of utterances by the interviewer and the interviewee. For instance, the interviewer asks the same question multiple times by rephrasing it to derive meaningful responses from the patient. Therefore, the answer to a question at the beginning of the interview can be found at the end or later parts of the interview (ie, anaphora [44]). To address this issue, we converted questions and answers into a template “participant was asked X, the participant said Y” so that TextRank can measure the statistical relevance of the response to the question (if the answer is contextually irrelevant to the question, the TextRank algorithm fails to generate a cohesive graph; rather, it generates disjoint graphs—one for the question, one for the answer—with no common node). Although we optimize using ILP over such disjoint structures, the informativeness score is still very low, as distances are large (a constraint that we minimize in ILP). Therefore, it is essential to develop a summarization model to recognize this structure.

We recognize that, in some cases, this structure might generate longer statements; however, the statement conveys minimal information. There are various forms of AS, which include or exclude paraphrasing, depending on the problem. For this study, we built and improved upon the past work on AS from Banerjee et al [38], Filippova [19], and Tuan et al [44]. In our qualitative evaluation guided by our domain expert, we specifically focused on the ability of the model to select good questions and meaningful responses.

### Baseline Summarization Methodologies

We considered 3 baselines for comparison with the proposed approach. First, ES generates human-readable summaries. The greedy nature of ES identifies meaningful utterances from the interview scripts. Second, we use AS, which brings coherence to the summaries. Third, we hybridized *abstraction over extractive summarization* (AoES) to leverage the advantages of ES to improve AS summary quality.

#### Extractive Summarization

ES generates a subset of sentences from the input document of the corpus as a summary. This approach can be likened to a condensation of the source document according to the *what you see is what you get* paradigm. ES techniques generally guarantee the linguistic quality of the generated summaries, whereas they are not abstractive enough to mirror manual summaries. We used the SumBasic (SB) algorithm to perform ES over the interview transcripts [45]. SB selects important sentences based on word probabilities (P(w_i_)) [46]. For a given input sentence (S_j_), the sentence-importance weight is computed using Equation 1:



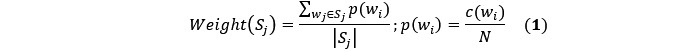



where c(w_i_) is the number of occurrences w_i_ in the input sentence, and N is the total number of words in the input sentence (N>>S_j_). A greedy selection strategy of SB selects the sentence that contains words with the highest probability. This selection strategy embodies the intuition that the words with the highest probabilities represent the document’s most important topics. After selecting a sentence, the word probabilities in the selected sentence are updated by squaring word probabilities before the sentence was selected. Such an SB update prevents the selections of the same or similar sentences multiple times, thus creating a diverse sentence summary. However, ES-generated summaries, although short, lack readability and informativeness. These are critical criteria in making understandable abstracts of clinical interviews where understandability takes precedence over summary length.

#### Abstractive Summarization

Typically, in a clinical diagnostic interview, the patient’s response aligns better with the question asked earlier in the interview. Therefore, to obtain a meaningful summary, it is necessary to consider the informativeness of a response to the question that has been asked earlier. Relating the answer to the most relevant question improves the cohesiveness of the summaries. This thematic rephrasing aspect makes AS superior to ES [47,48]. We implemented the AS method using the ILP optimization framework to optimize linguistic quality and informativeness by leveraging a generic LM [22]. This study contrasts with existing studies investigating supervised AS algorithms leveraging sequence-to-sequence architectures because we do not rely on human-written summaries [49]. A caveat in the use of AS in an unsupervised setting is its susceptibility to generate summaries with inaccurate information because the constraints do not consider domain knowledge. To minimize the impact of unsupervised learning, particularly in the mental health domain, constraints can be modeled using medical domain knowledge (eg, UMLS [Unified Medical Language System] and International Classification of Disease, 10^th^ Edition) to generate outcomes that facilitate reliable decisions to develop our proposed approach, KiAS.

#### Abstraction Over ES

AoES uses ES as a prefilter so that AS can generate high-quality summaries. However, AoES fails to focus on the domain-specific verbiage implicit in the conversation. For example, in the following conversation piece, “participant was asked what is going on with you, a participant said i am sick and tired of losses,” the italicized phrases are essential to an MHP but occur with low frequency and thus get removed by AoES. Another example, “participant was asked that's good where you are from originally, the participant said originally I’m from glendale california,” was identified as relevant by AoES. In contrast, it was filtered out using the proposed approach. Although the location in which people live could influence their mental health [50], it is less of a concern in clinical diagnostic interviews, which are face-to-face.

#### Proposed Approach: KiAS

**Figure 2:**
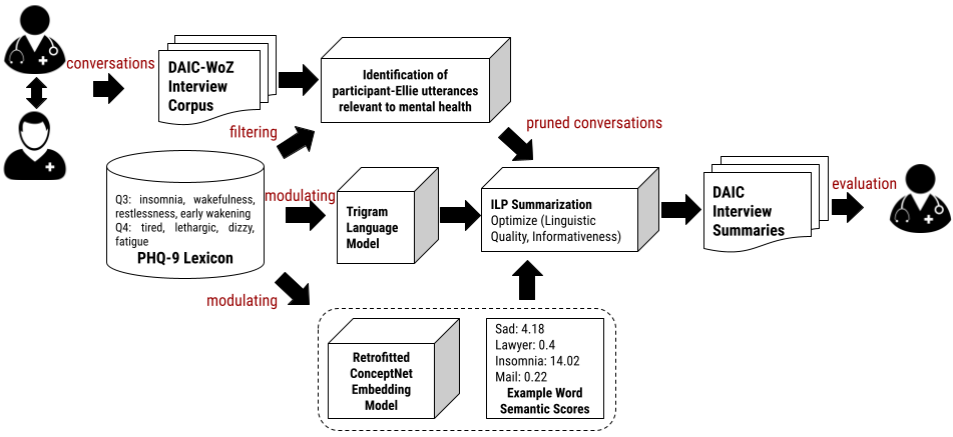
The overall workflow of our proposed model to generate contextual summaries of clinical diagnostic interviews. First, a PHQ-9 lexicon filters out irrelevant occurrences. Next, the ConceptNet embedding model was modulated using PHQ-9 Lexicon using a retrofitting procedure. The improved model was used to generate Word Semantic Scores. These scores quantify the importance of words in a conversation piece. A unified ILP framework of pruned conversations, trigram language model, and WSS, generates abstractive summaries. Q3 and Q4 in PHQ-9 Lexicon are questions in the PHQ-9 questionnaire. So, our Lexicon has nine categories associated with nine items in the questionnaire. DAIC-WoZ: Distress Analysis Interview Corpus-Wizard of Oz; ILP: integer linear programming; PHQ-9: Patient Health Questionnaire-9.

KiAS has 4 key steps: (1) creation of pruned conversations, (2) tuning generic LM, (3) retrofitting the concept net embedding model, and (4) creating abstractive summaries in an unsupervised manner. Figure 2 illustrates the proposed framework of KiAS for generating knowledge-aware summaries from real-world, simulated clinical diagnostic interviews in the DAIC-WoZ data set.

##### Creation of Pruned Conversations

In the clinical diagnostic interview, the interaction between the patient and MHP is initially unstructured and noisy but becomes focused and specific to mental health over time. The interview involves exchanging medical analogies and condition-expressive phrases, which implicitly refer to mental health conditions. These phrases were identified using semantic lexicon. Furthermore, to improve the quality of the summaries, initial filtering of noninformative summaries needs to be conducted.

A cleaned set of conversations is termed as *pruned conversations* and is essential to allow KiAS to put more weight on terms that are important to MHPs. In this pruning process, we used the PHQ-9 lexicon [34], which covers concepts related to depressive disorders obtained from human-curated medical knowledge bases (eg, UMLS and SNOMED-CT) and web dictionaries (eg, Urban Dictionary). We extracted phrases (eg, bigrams and trigrams) from the pruned conversations and find their presence in the PHQ-9 lexicon. We used normalized pointwise mutual information (NPMI) to evaluate the quality of n-grams. On the basis of the observation that trigrams NPMI scores (0.78) are higher than those for bigrams (0.72), we used a trigram LM in our summarization process [51].

##### Tuning the Generic LM

Our primary motivation for using an LM is to improve the linguistic quality of generated summaries. LM implicitly measures the grammatical nature and readability of text. On the other hand, one can either use a pretrained generic LM as-is or tune an LM for a specific domain and task. In this study, we chose to optimize an existing generic LM (we used a trigram model from CMUSphinx [52]) using the PHQ-9 lexicon.

This ensures that depression-related terms are retained when sentences are synthesized. We introduced a semantic score for each word in the sentence, termed the word semantic score (WSS), to measure how a word relates to the depression symptoms present in the PHQ-9 lexicon. To compute this semantic score, we leveraged ConceptNet embeddings retrofitted with the PHQ-9 lexicon [53].

##### Retrofitting ConceptNet

This procedure enriches word representations in the generic word-embedding model using a semantic lexicon [53]. In this study, we retrofitted ConceptNet to improve the similarity between the concepts of clinical relevance using the PHQ-9 lexicon. For example, the similarity between the words *feeling* and *lethargic* is 0.39 in ConceptNet, whereas after retrofitting, it is 0.86. As generic LMs generate representations of words in a sentence solely depending on the data, they may not reflect the true meaning of these words. Retrofitting adjusts the representations of words related to depression to have a similarity score to those in the lexicon. With the retrofitted ConceptNet embeddings, a word’s semantic score is the maximum cosine similarity the word can have with a term in the depression lexicon. The formulation for WSS is as follows:



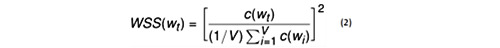



Here, *{c(w_t_) = max_w_k_^l^_ cos(w_t_,w_k_^l^)│ l ∈ lexicon categories, k ∈ l}*, denotes the maximum cosine similarity of w_t_ with a term in the depression lexicon. WSS improves the linguistic quality of the summaries by enhancing the probabilities of trigram phrases.

##### Knowledge-Infused Abstractive Summarization

We input the semantic score of a word, the trigram LM, and the relevant utterances to the ILP framework that will maximize the summaries’ informativeness and linguistic quality (Table 1). In the context of clinical diagnostic interviews, where the presence of anaphora and the free-flowing nature of discourse is profound, a Q and A pair would carry more meaning if previous and subsequent utterances were examined. For example, the Q and A pair—Ellie: “Was that hard for you?” Participant: “Yes”—can be unambiguously decoded if the previous few interactions between the interviewer and the participant are used to provide the necessary context for interpretation. Our domain experts estimated 7 Q and A pairs as an adequate window to capture the context based on a random sample of 25 patient conversations. The pruned conversations were divided evenly into the maximum number of slices such that each piece was not larger than 7 Q and A pairs. For example, a pruned conversation with 20 Q and A pairs is divided into a set of 3 slices (S) of sizes 7 (s_1_), 7 (s_2_), and 6 (s_3_) Q and A pairs. Furthermore, these fixed-window slices enable grouping the most semantically related sentences, which enhances informativeness.

**Table 1:** Example dialogues with their respective informativeness (I) and linguistic quality (Q) scores.

Example question and answer pair (path in word graph)^a^	Informativeness score (I)	Linguistic quality score (Q)	Included in summary
Participant was asked have they been diagnosed with depression participant said yeah while ago	0.25	0.1	Yes
Participant was asked uh huh, then participant said pretty easy	0.08	0.04	No

^a^Sentences containing words that are semantically close to those in the depression lexicon have higher Q than those that do not. The last column says whether the integer linear programming framework selected the sentence or not based on I and Q.

###### Informativeness (I)

**Figure 3:**
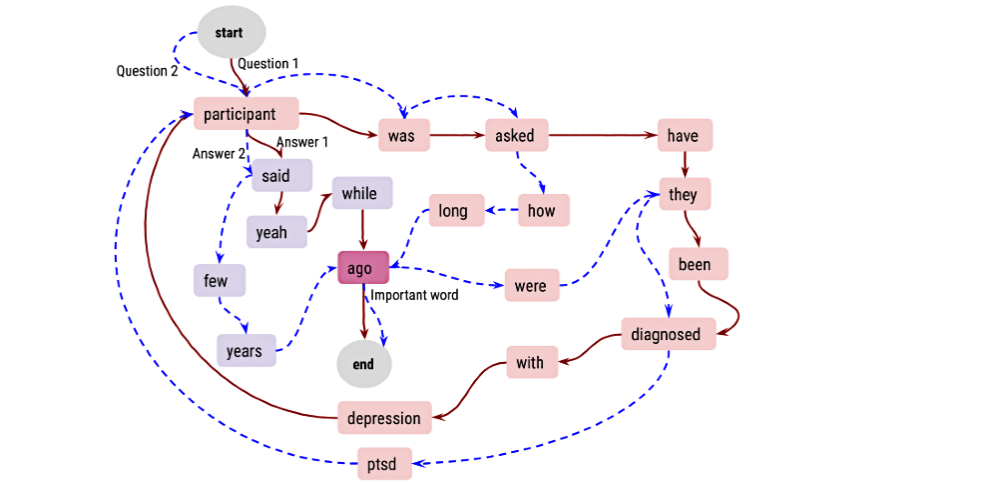
Word Graph (WG) of two Q/A pairs from the Distress Analysis Interview Corpus-Wizard of Oz data set. WG shows “ago” and “diagnosed” as the words with high importance score. WG is created per patient interview and becomes dense as the interview proceeds. This allow the summarizer to locate important words. However, importance scores of domain-specific words “depression” and “ptsd” is elevated using the word semantic score. Start and End are dummy nodes. ptsd: posttraumatic stress disorder.

We used TextRank that creates a WG from these slices, with words as vertices along with their scores computed based on in-degree and out-degree metrics (Figure 3) [20]. The frequency of connections between words in a corpus determines the contextual information, thereby factoring in informativeness [54]. TextRank assigns higher scores to vertices (words) with higher degrees in WG. The score of a given vertex v_i_ in WG is calculated as follows:



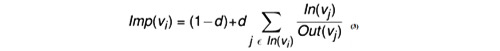



where the damping factor *d* is set to 0.78 (defined empirically), In (v_i_) denotes the in-degree of v_i_, and Out (v_j_) denotes the out-degree of v_j_. The information content of a path in a slice *I(p_i_^s_j_^)* is calculated as the sum of its words’ scores (Imp(v_i_)) that represent their importance.

###### Linguistic Quality (Q)

Sequential characteristics (ie, using an LM) and contextual coherence (ie, via the WSS of the words in the path) determine the linguistic quality of a sentence. Our approach uses the contextual knowledge using WSS (defined in Equation 2) in the LM, emphasizing the sentences with more frequent trigrams and words extracted through the mental health lexicon. The modified Q of a path p_i_ in a slice s_j_ is defined as:



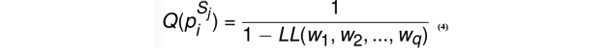





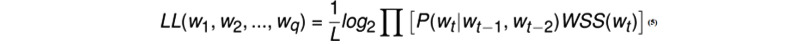



where p_i_ is the i^th^ path in the WG formed from q words (w_1_, w_2_,...,w_q_). L is the number of conditional probabilities; (P (w_t_ ǀ w_t−1_, w_t−2_)) is calculated using the words in p_i_, (w_1_, w_2_,...,w_q_). 1−LL (log-likelihood) in Equation 5 maximizes the understanding of the summaries.

###### ILP Formulation

To simultaneously maximize both Q and I of s_j_, we formulated the following objective function:



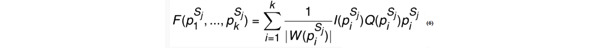



The ILP framework optimizes *I(p_i_^s_j_^)* and *Q(p_i_^s_j_^)* of a path *p_i_* and does the same over K paths in the slice *s_j_(s_j_ ⊂ S)* created from a pruned conversation. The term 
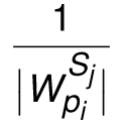
 ensures that higher weight is given to paths with fewer words for concise summary generation, where *│W(p_i_^s_j_^)│* indicates the number of words in the path. To ensure that F chooses one path from WG of paths *({p_1_, p_2_,...,p_K_} ⊂ s_j_)*, it adheres to the following constraint:

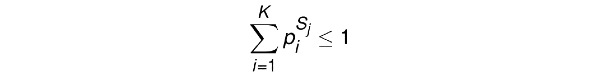



## Results

### Extrinsic Evaluation

**Figure 4:**
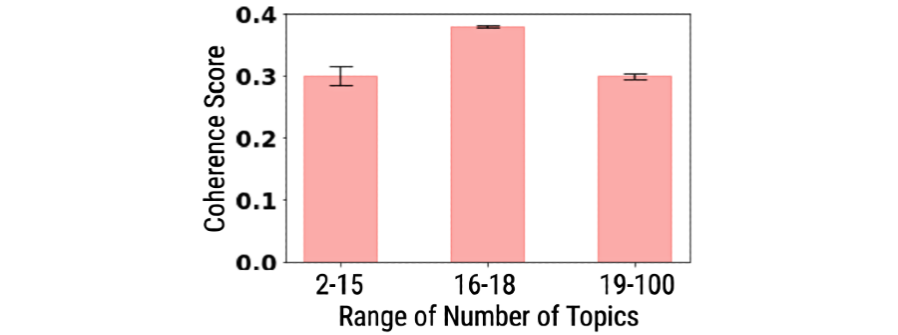
Coherence scores of latent Dirichlet allocation trained over pruned conversations (or reference summaries) over a number of topics. The topic coherence was measured using the formulation of CV [55].

As there are no ground truth summaries of the clinical diagnostic interviews for our task, we considered the pruned conversations (which are the inputs that need to be summarized) as if they were written by humans as reference summaries (RS). For an extrinsic evaluation of the summaries, we adopted JSD, contextual similarity, Flesch Reading Ease (FRE), and Topic Coherence Scores [55]. JSD measures how well the generated summaries probability distribution approximates the reference probability distribution. We considered the RS to provide the actual probability distribution over mental health concepts (input) and the generated summary as providing an approximation. For JSD, we needed to represent the generated summaries and RS. For this, we trained a topic model and used it to create a probabilistic topical representation of the generated summaries and RS. We trained Latent Dirichlet Allocation (LDA) topic models with varying numbers of topics (2 to 100) and measured coherence scores for each model. From Figure 4, a trained LDA model with 18 topics, having the highest coherence score, was used to generate topical representations of reference and generated summaries. For contextual similarity, we used the cosine-similarity metric to measure the distance between representations of the generated summaries and RS using retrofitted ConceptNet embeddings. A higher contextual similarity score is desirable. We used FRE to measure the readability of the text generated by an automatic summarizer [20]. A higher score reflects the ease of reading. Note that FRE is a validated instrument in the domain of education. Although generated summaries are expected to contain more focused and domain-specific information, they should have a considerable thematic overlap with the pruned conversations (or RS). Topics that describe psychological distress (eg, “I was diagnosed with ptsd a couple of weeks back”) and multiple behavioral dimensions (eg, sad, anger, positive or negative empathy, feeling low) need to be captured in summaries.

We used the LDA model trained over pruned conversations to create topics of summaries generated from different summarization methods. The thematic overlap score was calculated for the generated summaries [56]. We expected the created summaries to provide information on the diagnostic disorder (eg, stress and depression), patient behavior (eg, sad, feeling lonely, and lack of sleep), and time-related information (eg, years, ago, and weeks). As expected, a higher score for thematic overlap was desired.

### Extrinsic Evaluation Results

The interviews transcribed in DAIC-WoZ are diverse and noisy discourses because of poor grammar, thereby making the inputs significantly challenging to interpret across different patients. Moreover, some of the patients’ responses were short, with no mental health–related information. We showed these variations and linguistic irregularities using the metrics of contextual similarity, JSD, readability, and thematic overlap across 184 patients and reported mean scores with SDs for comparison.

**Figure 5:**
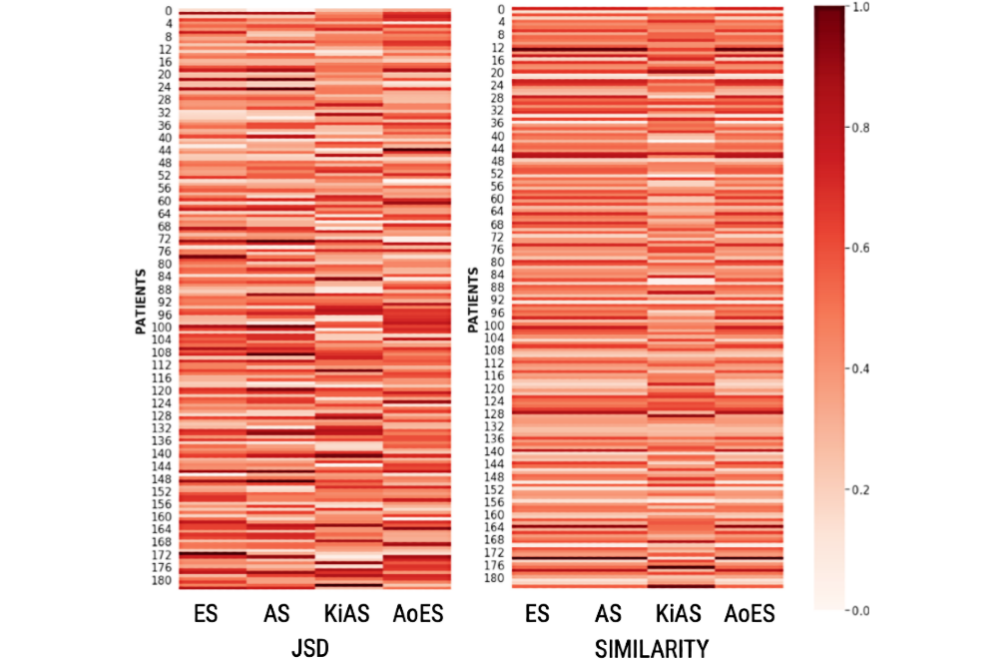
The heatmap shows the evaluation of generated summaries using information entropy and contextual similarity metrics. The figure shows the performance of KiAS against three baselines on retaining the content and context in summaries. AoES: abstraction over extractive summarization; AS: abstractive summarization; ES: extractive summarization; KiAS: knowledge-infused abstractive summarization.

**Figure 6:**
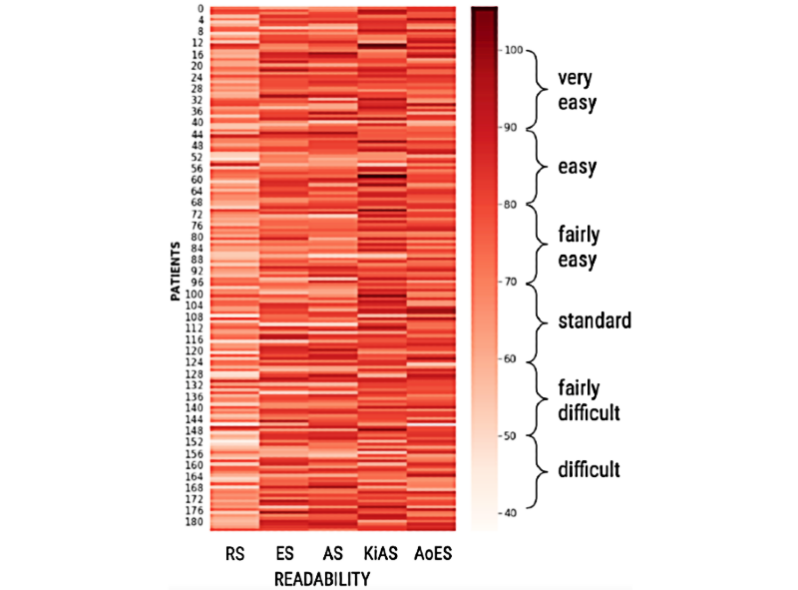
Heatmap of 184 patient summaries created using the Flesch Reading Ease scale. We compare the readability of summaries created from ES, AS, AoES, and KiAS with reference summaries. Darker patches indicate patient summaries, which are easy to read. AoES: abstraction over extractive summarization; AS: abstractive summarization; ES: extractive summarization; KiAS: knowledge-infused abstractive summarization; RS: reference summaries.

**Figure 7:**
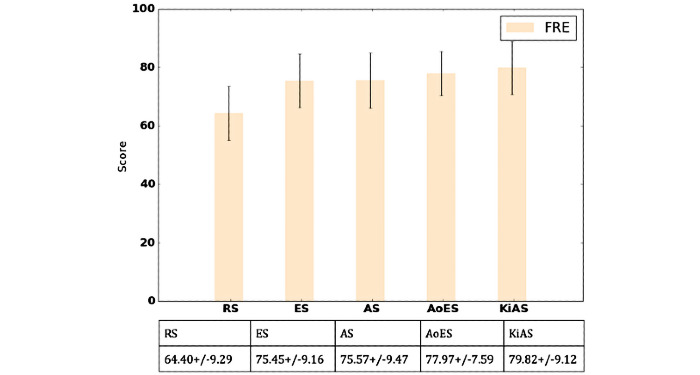
Evaluating readability of the generated summaries from RS. FRE method assesses comprehensibility and engagement of the summaries. AoES: abstraction over extractive summarization; AS: abstractive summarization; ES: extractive summarization; FRE: Flesch Reading Ease; KiAS: knowledge-infused abstractive summarization; RS: reference summaries.

In Figure 5, we compare the summaries’ representations of each patient with the averaged representation of the RS. We observed that the summaries generated by ES, AS, and AoES, were similar, whereas those produced by KiAS were marginally better. We used information entropy (JSD) to measure the information gain in the summarization approaches (Figure 5 and Table 2). A lower JSD was more desirable. We observed that the JSD of KiAS was relatively small (ES [2.5% decrease], AS [2% decrease], and AoES [3% decrease]), and 2.2% decrease on average, as the use of the PHQ-9 lexicon and retrofitted ConceptNet enables KiAS to preserve the semantic relationships in the dialogue. For example, “Have you been diagnosed with clinical depression?” and “How often did you feel depressed because of poor sleep and fatigue?” elicit similar information from the patients. The response to either of these questions is related to the mental condition; therefore, it should be informative and preserved. Figures 6 and 7 show a comparison between the baseline summarization approaches and KiAS for readability. We observed that KiAS provides better readability than the baseline methods (ES [5.5% increase], AS [5.3% increase], and AoES [2.3% increase]), whereas all summaries seem to be more readable than the pruned conversations.

**Table 2:** Evaluating contextual similarity (higher is better) and information entropy (lower is better) of summaries generated from four different summarization approaches. The reported scores are mean and standard deviation calculated across 184 patient conversations in the Distress Analysis Interview Corpus-Wizard of Oz data set.

Methods	Contextual similarity, mean (SD)	JSD^a,^ mean (SD)
Extractive summarization	0.672 (0.076)	0.387 (0.107)
Abstractive summarization	0.676 (0.076)	0.373 (0.102)
Abstractive over extractive summarization	0.669 (0.092)	0.385 (0.103)
Knowledge-infused abstractive summarization	0.689 (0.088)	0.360 (0.104)

^a^JSD: Jensen Shannon Divergence.

We measured the thematic overlap between the referenced and generated summaries to assess the presence of psychological stressors and behavioral signals, which would be helpful for MHPs. We noticed that ES, AS, and AoES created summaries capturing time-related words (eg, how long, away for a while, about a year, end of the day) and emotional words (eg, annoyed, obnoxious behavior, happy, angry); however, disorder- and response-related words were rarely present. KiAS summaries showed higher occurrences of psychological stress-related words (eg, lack of energy, dizzy whole day, loss of appetite) and behavioral concepts (eg, down in guilt and fear of returning to work). As a result, we observed ~40% thematic overlap between KiAS and reference summaries, which is 17%, 20.5%, 32.5% better than ES (32.8%), AS (31.5%), and AoES (26.65%). On the other hand, KiAS might provide low-quality summaries when the conversation is introductory and does not use mental health-specific words (white and dark patches in Figures 5 and 7).

In comparing KiAS with ES based on FRE, JSD, and contextual similarity scores, we noticed a significant statistical difference on a two-tailed *t* test at a significance level of .05. Across all 184 patients’ summaries, which were quantitatively and qualitatively evaluated, KiAS gave consistently higher scores than ES. Similarly, KiAS summaries were reasonably statistically significant when compared with AS and AoES across the various FRE, JSD, and contextual similarity scores. The main feature of KiAS is to reveal the implicit clinical information from the interviews, specifically the responses of patients to the questions; however, there are few such conversations. Therefore, noticeable differences in KiAS summaries can be observed by focusing on these samples. Nonetheless, we observed an improvement over AoES and AS, although it was modest.

### ROUGE Evaluation

ROUGE is a measure of the informational adequacy of the summaries generated from a summarization system given as input as long text (eg, paragraph, meeting notes, interview logs, or RS). The metric is statistical, as it measures the overlap in n-grams in the generated summaries and RS. We reported *F*_1_ scores and Recall of KiAS and compared it with other methods of summarization (ES, AS, and AoES, Table 3). As KiAS and AS methods are guided by constraints, we considered recall as an appropriate measure, alongside the *F*_1_ score for performance evaluation. With an improvement of approximately 61% in ROUGE-2 (ROUGE for bigrams) recall, KiAS captures more relevant bigrams compared with ES and AS. Most of the clinical terms in mental health care occur as bigrams, and the use of the PHQ-9 lexicon (which contains mostly bigram phrases) enabled the generation of comparatively more relevant summaries (approximately 55% improvement in the *F*_1_ score). In another version of ROUGE-2, ROUGE-L (ROUGE for longest subsequence) calculates the sentence-level structural similarity between the generated summaries and RS. KiAS outperformed ES and AS and AoES with improvements of 48%, 48%, and 53%, respectively, in ROUGE-L recall. We did not see bilingual evaluation understudy as an appropriate metric based on the reasons mentioned in previous literature [57,58]. We further conducted a human evaluation to analyze the informativeness and fluency (or readability) of the generated summaries [58,59].

**Table 3:** Quantitative Recall-Oriented Understudy for Gisting Evaluation (ROUGE)-1, ROUGE-2, and ROUGE-L results on the summarization task of clinical diagnostic interviews. All the scores have a 95% CI of at most ±0.18 (SD).

Methods	ROUGE^a^-1			ROUGE-2			ROUGE-L	
	Recall	*F*_1_ score	Recall		*F*_1_ score	Recall		*F*_1_ score
KiAS^b^	22.53	30.43	10.65		14.62	24.46		32.57
Abstractive summarization	9.89	15.22	4.09		6.42	12.80		20.53
AoES^c^	8.69	12.37	2.25		3.21	11.51		17.24
Extractive summarization	9.79	14.37	4.18		6.52	12.72		20.47

^a^ROUGE: Recall-Oriented Understudy for Gisting Evaluation.

^b^KiAS: knowledge-infused abstractive summarization.

^c^AoES: abstraction over extractive summarization.

### Intrinsic Evaluation

Our qualitative evaluation has been designed by 4 practicing psychiatrists who are also the end users of the proposed system. We evaluated the quality of the questions and meaningful responses provided by the patient. Our rubric for domain expert evaluation was as follows:

1. Good question with unclear context: the summary may or may not include context related to mental health, specific to the patient. For example, the patient was asked, “When was the last time that happened?” for which the referent of that is unclear.
2. Good question with clear context: the summary includes context related to only the mental health situation of the patient. These questions are complete, and no inference is required by MHPs. For example, the patient was asked, “Did you ever suffer from PTSD?”
3. Meaningful response: the response is meaningful and understandable concerning the patient and the question asked by the MHP. For example, the patient was asked, “Have you ever been diagnosed with depression?” The patient then responded, “Not really,” meaningful only in the context of the preceding question.

Another example: a patient was asked, “How long ago were you diagnosed?” The patient responded, “A few years ago.” In this qualitative evaluation, we only considered summaries generated from KiAS and AS because ES- and AoES-based summaries were not identified as useful by our collaborator MHPs. Randomly selected 25 (179 Q and A sentences) patient summaries were given to the 4 practicing MHPs for expert evaluation. In summary, if a sentence is (1) a good question with an unclear context, (2) a good question with a clear context, or (3) a meaningful response, a +1 score is given. A score of 0 is assigned otherwise. We then totaled the scores for each patient. Considering the varied experiences of MHPs in treating patients, we investigated interrater agreement using Cohen κ [60,61].

### Intrinsic Evaluation Results

To evaluate the quality of summaries from the clinicians’ perspective, we randomly selected 25 summaries (179 Q and A pairs) generated from AS and KiAS methods. For intrinsic evaluation, MHPs would first read the original interview transcript of the patient and then assess the summaries based on (1) the number of good questions and (2) the association of the most relevant patient’s response to the good questions.

Overall, KiAS (1) provides more contextual questions and answers, (2) is more informative even in the absence of context, (3) captures implicit references to medical vocabulary in patients’ answers, and (4) improves upon AS on identifying good questions by 2.6% and meaningful responses by 4.1%. Better Q and A with meaningful responses should improve the follow-up time for MHPs. The 4 MHPs had an estimated reduction of up to 46% in the follow-up time with KiAS than without KiAS, enabling them to see more patients. MHPs mostly agree on their evaluation of KiAS and AS, although MHP 3 is more inclined toward AS than KiAS (Table 4). MHPs provided a substantial agreement that both KiAS and AS provided *good questions*, whereas they showed substantial agreement on KiAS for *meaningful response* compared with a moderate agreement on AS (Table 5).

**Table 4:** Performance of methods on intrinsic evaluation from 4 mental health professionals gathered after counting the number of good questions with clear context, good questions with unclear context, and meaningful responses in 50 (2 methods) summaries generated.

Domain Experts	MHP^a^ 1			MHP 2			MHP 3			MHP 4	
Methods	AS^b^	KiAS^c^	AS		KiAS	AS		KiAS	AS		KiAS
GQCC^d^	71	78	75		74	66		63	59		64
GQUC^e^	86	92	93		95	87		85	82		85
Meaningful responses	60	63	61		65	52		57	72		76

^a^MHP: mental health professional.

^b^AS: abstractive summarization.

^c^KiAS: knowledge-infused abstractive summarization.

^d^GQCC: good questions with clear context.

^e^GQUC: good questions with unclear context.

**Table 5:** Interannotator agreement (Cohen κ) calculated over summaries evaluated by 4 mental health professionals.

Method	Good questions^a^, Cohen κ	Meaningful response, Cohen κ
Abstractive summarization	0.63	0.43
KiAS^b^	0.70	0.65

^a^Good questions include good questions with unclear context and good questions with clear context.

^b^KiAS: knowledge-infused abstractive summarization.

## Discussion

### Principal Findings

Textbox 2 shows summary snippets generated from AS and KiAS for patient ID 313 (because of a page limit, we have not shown the pruned conversation of patient ID 313, but it can be viewed on a link [62]. The number of Q and A pairs are 51 (number of words=1190). On visual inspection of the summaries by 4 MHPs, we found that the summary provided by the KiAS was more informative and aligned with relevant responses to relevant questions. For example, considering the patient question “How long ago were they diagnosed with depression?” the response obtained by KiAS was “a year ago,” which is meaningful compared with the response by AS (“they are still depressed”).

Furthermore, an implication that can be drawn from Table 4 is that our summary includes the reason for the patient’s visit, which makes questions on *therapy* and *diagnosis of depression* relevant. However, the *purpose of the visit* is missing in the summary generated by AS. Although the summary created from our approach is longer than that from AS, a recent study by Sun et al [63] illustrated that summary length alone is not a good measure of the summary. To validate the quality of KiAS, we performed an intrinsic evaluation designed to investigate its potential utility in real-world applications.

**Summary generated using abstractive summarization and knowledge-infused abstractive summarization. The proposed approach captures near-exact questions and responses as in Distress Analysis Interview Corpus-Wizard of Oz (DAIC-WoZ) interview scripts compared with AS. Both models received the same pruned conversations of patient ID 313 from the DAIC-WoZ. The tendency to model Q and I as constraints in integer linear programming enhances its capability to generate descriptive summaries.**

**Summary using abstractive summarization**

- *Participant was asked*: What do they do when they are annoying until they stop
- *Participant said*: That they stop talking
- *Participant was asked*: When was the last time they felt really happy
- *Participant said*: A year while ago
- *Participant was asked*: How long ago were they diagnosed depression
- *Participant said*: They are still depressed

**Summary using knowledge-infused abstractive summarization**

- *Participant was asked*: What do you do when they are annoying
- *Participant said*: She stop talking
- *Participant was asked*: Can you explain with example
- *Participant said*: Yeah
- *Participant was asked*: When was the last time they felt happy
- *Participant said*: A while ago
- *Participant was asked*: What got them to seek help
- *Participant said*: They are still depressed
- *Participant was asked*: Tell me more about that
- *Participant said*: Yeah
- *Participant was asked*: Do they feel like therapy useful
- *Participant said*: Oh yeah definitely
- *Participant was asked*: How long ago were they diagnosed depression
- *Participant said*: A year ago

### Conclusions

We experimented with the infusion of knowledge to summarize mental health conversations that contain ambiguous, noisy, and nongrammatical content while preserving the key low-frequency content of clinical significance. Using clinical diagnostic interviews, we proposed a simple and effective summarization strategy called KiAS, which incorporates the knowledge in the PHQ-9 lexicon into an ILP framework to extract and summarize Q and A pairs that describe a patient’s condition. We used a sequence of understandable evaluation criteria to test the summarization framework’s ability to capture contextual, syntactic, and semantic characteristics that match human-level judgments. Our approach significantly outperforms the baselines on dialogues that had explicit and implicit indicators of mental health conditions. Furthermore, our evaluation showed substantial agreement between MHPs regarding the presence of *good questions* and *meaningful responses* in summaries.

### Limitations and Future Work

The result of this study is to inform the development of an automated summarization tool for clinical diagnostic interviews. The clinical interviews were characterized by open-ended conversations comprising questions on diagnosis, symptoms, medication, and response, which provide implicit references to disorders, symptoms, and medicine. Our analysis of the interview transcripts showed a minimal number of medical concepts, redundancy in questions and responses, and implicit references to mental health conditions. These data characteristics raise challenges in the summarization task in addition to data sparsity because of the limited number of patient interviews. Our primary goal was to demonstrate the feasibility of a method that generates actionable summaries containing meaningful questions and responses for a well-informed follow-up. With guidance from coauthor MHP, we selected the ILP framework, which allows integration of domain knowledge to resolve summarization over clinically sparse data, optimize informativeness in summaries, and generate summaries with good linguistic quality. By optimizing *informativeness* using the PHQ-9 lexicon, our method elicits cues that act as subtle indicators of human emotions. However, we certainly acknowledge that abstractive systems may lose nuance from their rewriting of the text and accept this trade-off to improve recall or reduce information overload. Similarly, social media interaction typically demonstrates sarcasm, irony, and other idiomatic languages that are difficult to capture using deep-learning approaches. Thus, we state that “an accurate assessment, diagnosis, and treatment, hinges on the ability of a trained MHP to elicit not only information but also extract subtle indicators of human emotions that portend clues to a severely life-threatening situation” with a caution that generated summaries need to be evaluated by domain experts.

A limitation of this study is that we did not explore transformer-based architectures (eg, Medical Bidirectional Encoder Representations from Transformers, Clinical Bidirectional Encoder Representations from Transformers, and BART), fine-tuning procedures, and pretrained models owing to (1) the lack of sufficient patient interviews, (2) no gold standards, and (3) the lack of data from discussion forums that parallel the structure of clinical interviews. Aside from the limitations on the data sets and insufficient exercise of deep-learning methods, a different set of MHPs may have highlighted a different approach or qualitative evaluation strategy. Our research was limited to transcripts from the University of Southern California Institute of Creative Technologies.

Furthermore, we are working toward extending the data set cohort by including clinical interviews recorded by clinicians in these medical schools. This sample may not represent diagnostic interviews in other clinics, such as the University of California, Los Angeles; University of California San Francisco; Weill Cornell; and Wake Forest School of Medicine, which follow different styles of eliciting information from patients. This can be remedied in future studies by having MHPs at these medical schools. Furthermore, we would extend our qualitative analysis of opinions from MHPs from these medical schools. Furthermore, we envisioned that our approach can be used with guidance from an MHP in a mental health virtual assistant (VA) app to summarize the conversations between the patient and VA.

### Reproducibility

We acknowledge the DIAC-WoZ dataset created by the Institute for Creative Technologies at the University of Southern California is available at: . The repository containing the models, including the baselines, is made public on: .

## Abbreviations

AoES: abstraction over extractive summarization
AS: abstractive summarization
BART: Bidirectional and Auto-Regressive Transformers
ES: extractive summarization
FRE: Flesch Reading Ease
ILP: integer linear programming
JSD: Jensen Shannon Divergence
KiAS: knowledge-infused abstractive summarization
LDA: Latent Dirichlet Allocation
LM: language model
MHP: mental health professional
NIDA: National Institute on Drug Abuse
NIH: National Institutes of Health
NPMI: normalized pointwise mutual information
NSF: National Science Foundation
PHQ-9: Patient Health Questionnaire-9
PTSD: posttraumatic stress disorder
Q and A: question and answer
ROUGE: Recall-Oriented Understudy for Gisting Evaluation
RS: reference summaries
SB: SumBasic
SNOMED-CT: Systematized Nomenclature of Medicine-Clinical Terms
UMLS: Unified Medical Language System
VA: virtual assistant
WG: word graph
WSS: word semantic score
